# Acetate concentration correlates with MSNA in patients with resistant hypertension

**DOI:** 10.1007/s10286-025-01144-6

**Published:** 2025-07-17

**Authors:** Revathy Carnagarin, Gianni Sesa-Ashton, Natalie C. Ward, Janis Nolde, Anu Joyson, Justine Chan, Ancy Jose, Markus P. Schlaich

**Affiliations:** 1 Dobney Hypertension Centre, Medical School, Royal Perth Hospital Unit and RPH Research Foundation, The University of Western Australia, Level 3, MRF Building, 50 Rear Murray Street, Perth, WA 6000 Australia; 2 Cardiometabolic Health and Exercise Physiology Laboratory, Baker Heart and Diabetes Institute, Melbourne, Australia; 3 Department of Cardiology and Department of Nephrology, Royal Perth Hospital, Perth, WA Australia

**Keywords:** Short-chain fatty acids, Sympathetic nervous system, Microneurography, Resistant hypertension

## Abstract

**Purpose:**

Short-chain fatty acids (SCFAs), metabolites of colonic microflora fermentation of dietary fibre, have been implicated in experimental models and clinical trials to impact blood pressure (BP) regulation. Dietary interventions increasing serum SCFA levels have been associated with reduced 24-h systolic BP in hypertensive patients. However, the underlying mechanisms remain elusive. Given the role of the gut–brain axis and clear evidence for sympathetic nervous system activation as important modulators of blood pressure, we examined the relationship between sympathetic drive and SCFA concentration in patients with resistant hypertension (RH) and healthy control subjects (HC).

**Methods:**

A total of 21 patients with RH (68.6 ± 9.7 years, 47% male) and 28 healthy control subjects (HC) (34.6 ± 16.7 years, 75% male) were recruited to undergo microneurography for determination of muscle sympathetic nerve activity (MSNA), automated office BP (AOBP) and blood collection for serum SCFA.

**Results:**

Mean systolic AOBP was 156 ± 21 mmHg and 115 ± 10 mmHg for RH and HC, respectively (*p* < 0.0001). Serum acetate levels were 1340 ± 115.4 umol/L for HC and 724.5 ± 116.9 umol/L for RH (*p* < 0.0001). Butyrate and propionate concentrations did not significantly differ between groups. MSNA burst frequency was markedly elevated in RH compared with HCs (*p* < 0.001), with 25.3 ± 7.4 burst/minute in HC compared with 40.24 ± 8.3 burst/minute in RH. An inverse relationship was evident between serum acetate levels and MSNA burst frequency (*p* = 0.0267, *R*^2^ = 0.4) along with increased sympathetic vascular transduction (*p* = 0.0008, *R*^2^ = 0.82) in RH.

**Conclusions:**

Our findings suggest that the beneficial effects of SCFA levels, in particular acetate, on cardiovascular regulation may at least in part be mediated by sympatho-inhibition and altered sympathetic vascular transduction.

## Introduction

Hypertension remains the most important modifiable risk factor for cardiovascular morbidity and mortality worldwide, despite a host of effective lifestyle and pharmacological treatment modalities [1]. Patients with resistant hypertension defined as blood pressure that remains above recommended target levels despite the use of three or more antihypertensive drugs at maximally tolerated doses, are at increased risk of major adverse cardiovascular events [2] subserved by a multimodal pathophysiology involving sympathetic nervous system activation [3]. In patients with resistant hypertension, sympathetic drive measured either by noradrenaline spillover or muscle sympathetic nerve activity (MSNA) is markedly elevated relative to controls [4]. From a mechanistic perspective, it has been demonstrated that chronically elevated blood pressure leads to reduced capacity for the resistance vasculature to respond to increased sympathetic drive, typically quantified as sympathetic vascular transduction [5].

Recently, a potentially significant role of the gut microbiome has been postulated for the pathogenesis and maintenance of the hypertensive state. Diets high in fruit and vegetables are related to lower resting blood pressure [6, 7], with this effect at least partially mediated by increased dietary fibre intake—carbohydrates arriving from the diet to the colon undigested [8]. These dietary factors influence the dominant species within the gut microbiome [9] as well as drive the formation of short-chain fatty acids (SCFAs) through fermentation by resident bacteria of which acetate, butyrate and propionate are the most common and clinically relevant [10]. SCFAs have previously been shown to be cardioprotective in animal models. Acetate in particular has been shown to reduce blood pressure and prevent heart failure progression [11] while other SCFAs are implicated in platelet hyperreactivity and subsequent thrombotic risk [12]. Importantly, a recent double-blind clinical trial has shown prebiotic supplementation increased serum levels of acetate by shifting microbiome composition and reduced blood pressure in drug-naive hypertensive patients [13].

Mechanistically, SCFAs act on a host of systems and the means by which they may lower blood pressure in humans is poorly understood. Given the potential reductions in vascular resistance found in human studies [13], we sought to investigate whether there were apparent differences in circulating SCFA in controls and patients with resistant hypertension and whether MSNA and sympathetic vascular transduction correlated with SCFA concentrations in these groups.

## Methods

### Participants 

A total of 21 patients with resistant hypertension (RH; 68 ± 9.7 years, 47% male) and 28 healthy young controls (HC: 34.7 ± 16.7 years, 75.0% male) were recruited through the Royal Perth Hospital between 2017 and 2020 (Table 1). Healthy control subjects were recruited from the community. All participants with resistant hypertension were on three or more antihypertensive drug classes. Exactly 42% were prescribed angiotensin-converting enzyme (ACE) inhibitors alongside an additional 37% prescribed angiotensin-receptor antagonists. In addition, 84% were prescribed a calcium channel blocker, 25% were prescribed a beta blocker and only 3% prescribed an alpha-1 receptor antagonist. Finally, 84% were prescribed a thiazide-like diuretic, with no participants on loop diuretics. Participants remained on their typical antihypertensive therapy, with those on sympatholytic therapies, namely moxonidine or methyldopa, excluded from this study.

**Table 1:** Participant characteristics of patients with resistant hypertension and healthy controls

	RH (*n* = 21)	HC (*n* = 28)	*p*-value
Age (years)	68.6 ± 9.7	34.7 ± 16.7	*p* < 0.0001
Male (%, *n*)	47, 12	75, 21	*p* = 0.22
BMI (kg/m^2^)	29.99 ± 6.8	25.21 ± 4.3	*p* = 0.12
AOBP SBP (mmHg)	156.2 ± 21.0	115.3 ± 10.4	*p* < 0.0001
AOBP DBP (mmHg)	76.21 ± 24.0	70.27 ± 5.8	*p* = 0.46
Heart rate (BPM)	64.8 ± 7.4	61.53 ± 5.5	*p* = 0.15
Acetate (umol/L)	724.5 ± 116.9	1340 ± 115.4	*p* < 0.0001
Propionate (umol/L)	81.98 ± 49.6	83.23 ± 9.0	*p* = 0.88
Butyrate (umol/L)	10.44 ± 3.0	10.53 ± 2.3	*p* = 0.89

*BMI* body mass index, *AOBP* automated office blood pressure

All participants provided informed, written consent. All studies were approved by the University of Western Australia, Royal Perth Hospital or East Metropolitan Health Service Ethics Committees and all participants provided written informed consent. Prior to the experiment, participants were advised to refrain from caffeine and nicotine. No participants were habitual nicotine users but were counselled regardless.

### Collection of serum samples

Serum samples were collected in ethylenediaminetetraacetic acid (EDTA)-containing tubes, which were immediately centrifuged following venipuncture as previously described [14]. Individual serum samples were immediately placed in −80 ℃ freezers. SCFA analysis was completed on frozen samples which had never been previously thawed. Samples were spiked with 5 nmol of ^13^C-sodium acetate and 5 nmol of 2-ethyl butyric acid for use as internal standards and homogenised with isopropanol. Samples were then centrifuged with 1 uL of supernatant collected and placed into a HP 6890 Series GC system with Agilent 5973 Network Mass Selective Detector set to splitless mode. Samples were separated on a 30 m × 0.25 mm i.d Stabilwax-DA column (Shmadzu) coated with 0.25 um-thick film. Helium was used at a flow rate of 1 mL per minute as carrier gas. Oven temperature was initially 90 °C, held for two minutes, and increased to 240 ℃ in 5 ℃ per minute increments. Samples were held at 240 ℃ for an additional two minutes. The quadrupole, mass spectrometry (MS) source and inlet were held at 150 ℃, 230 ℃ and 250 ℃, respectively. Volatile-free acid mix was used during the quantification of retention times and SCFA identification. SCFA concentrations, expressed as nanomoles per microlitre of serum, was calculated based on internal references from three or more replicates.

### Muscle sympathetic nerve activity recording

Microneurography was recorded from the common peroneal nerve, approximately 1 cm inferior to the fibular head, using tungsten microelectrodes (FHC, Bowdoinham, ME) recording multi-unit MSNA. Participants were laid supine with one knee flexed and supported with a vacuum pillow, which would be utilised as the recording site. One active electrode was inserted toward the common peroneal nerve with a reference electrode placed within the skin ~2 cm away from the recording site. Both electrodes and a ground electrode ran into an analogue head stage, sampling at 1000 Hz. Data were recorded via a PowerLab (ML 785/8SP; ADInstruments, Bella Vista, NSW, Australia) and visualised in LabChart (ADInstruments). MSNA signal was confirmed via Valsalva maneuver (10 s, ~20 mmHg)—subsequent increases in activity indicating electrode placement within a nerve innervating arteriolar smooth muscle.

### Blood pressure measurement

All participants underwent automated office blood pressure (AOBP) according to World Health Organisation (WHO) standards. Patients underwent three BP recordings following a five-minute baseline period, spread one minute apart from each other, with the average of the recordings reported as their individual blood pressure.

### Data analysis

Data analysis was completed in the Ensemble analysis environment (Elucimed, Wellington New Zealand). Quality of recordings were assessed for viability using an inbuilt cross-correlogram, which correlates MSNA bursts with electrocardiogram (ECG) r-waves, with strong coupling implying confirmation of MSNA. Six RH participants lacked a sufficiently long MSNA recording or lacked sufficient R-R correlation to analyse, and as such were excluded form further analysis.

Automated burst counts were completed within Ensemble following data aggregation. Burst counts were checked against manually calculated burst frequency and burst incidence from within LabChart (ADInstruments, Version 7.1.2.5). Baroreflex sensitivity analyses were processed as a linear relationship between burst incidence and diastolic blood pressure over 3 mmHg bins within Ensemble, with an *R*^2^ of 0.50 or greater to be considered valid [5]. Similarly, sympathetic vascular transduction was calculated as the linear relationship between relative MSNA burst size, based upon the largest burst within a recording window, and diastolic blood pressure between 6 and 8 cardiac intervals following the burst, as performed by Kobetic et al. [5]. This is to account for the conduction delay inherent to vascular changes downstream of MSNA, which reaches its maximal pressor response between 6 and 8 cardiac cycles. An *R*^2^ of 0.50 was also required for the data to be considered valid. Given this, five MSNA recordings in patients with RH and six in HC participants had insufficient recording periods while continuous blood pressure was actively recording—largely owing to device error and calibration—to be appropriately analysed and as such, have been excluded.

Statistical analysis was completed in Prism 10 for MacOS (GraphPad, Boston, USA). Direct comparisons between patients with resistant hypertension and healthy controls were performed using unpaired *t*-tests following normality testing by D’agoistino and Pearson’s test. Correlation analyses were performed using simple linear regression alongside Pearson’s test for the assessment of the strength of proposed relationships. All data is provided as mean ± SD.

### Data availability

Data used for this study can be made available upon reasonable request to the corresponding author.

## Results

### Patient characteristics

Automated office blood pressure was 115.3 ± 10 mmHg systolic for HC and 156.2 ± 21.0 mmHg systolic for RH (*p* < 0.0001). Diastolic blood pressure did not significantly differ between RH and HC (*p* = 0.46) at 76.2 ± 24.0 and 70.3 ± 5.8 mmHg, respectively. Patients with RH were significantly older at 68.6 ± 9.7 years relative to healthy controls at 34.7 ± 16.7 years (*p* < 0.0001). Body mass index (BMI) was numerically higher  in patients with RH but did not reach significance (*p* = 0.12).

Serum concentrations of acetate were 1340 ± 115.4 umol/L for healthy controls and 724.5 ± 116.9 umol/L for patients with resistant hypertension, representing a statistically significant difference between the groups (*p* < 0.0001; unpaired *t*-test). However, neither propionate (*p* = 0.89) nor butyrate levels (*p* = 0.88) differed between RH and HC.

### Autonomic function in healthy controls and patients with resistant hypertension

**Fig. 1:**
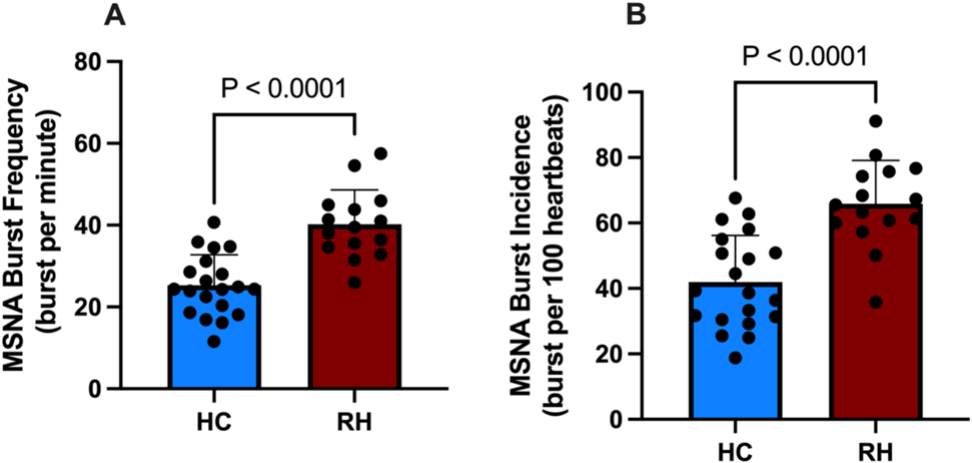
Burst frequency (**A**) and burst incidence (**B**) in healthy controls (HC, blue, *n* = 20) and patients with resistant hypertension (RH, red, *n* = 15). Data presented as mean ± SD. **** = *p* < 0.0001

Burst frequency, the number of bursts of MSNA per minute, was significantly higher in patients with resistant hypertension at 40.2 ± 8.4 bursts per minute compared with 25.3 ± 7.5 bursts per minute in healthy controls (*p* < 0.0001) (Fig. 1A). Similarly, burst incidence, the number of MSNA bursts per 100 heartbeats, was also significantly elevated in RHs compared with HCs (*p* < 0.0001) (Fig. 1B). Baroreflex gain was significantly reduced in RHs at −0.53 relative to −2.17 in HCs (*p* = 0.045). Sympathetic vascular transduction was not significantly different between RH and HCs (*p* = 0.72).

Correlation analyses between burst frequency in healthy controls revealed no significant correlations for acetate (*p* = 0.11), propionate (*p* = 0.35) or butyrate (*p* = 0.94). However, while considering burst incidence, both propionate (*p* = 0.74) and butyrate (*p* = 0.89) did not correlate; a moderate, positive correlation was apparent between burst incidence and acetate concentrations (*p* = 0.031) in healthy controls. Healthy controls showed no relationship between any SCFA concentrations and sympathetic vascular transduction (*p* > 0.2) or baroreflex gain (*p* > 0.4). Moreover, resting heart rate did not relate to SCFA concentrations in HC (*p* = 0.44).

**Fig. 2:**
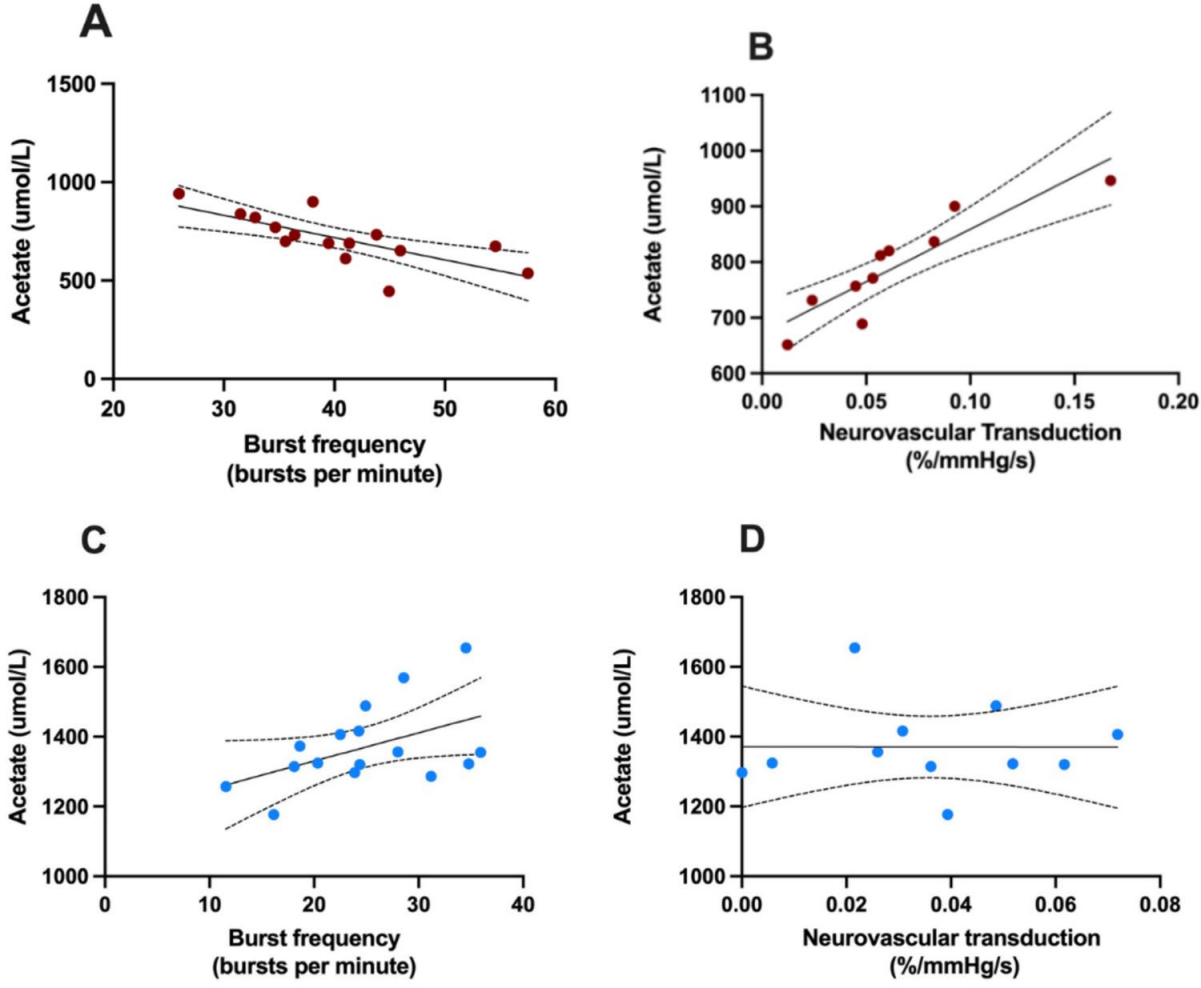
Simple linear correlation of acetate concentration with burst frequency (**A**) and sympathetic vascular transduction (**B**) in patients with resistant hypertension. Simple linear correlations between acetate concentration and burst frequency in healthy controls (**C**) as well as sympathetic vascular transduction (**D**) are also shown. Solid line indicates line of best fit. Dashed lines indicate 95% confidence interval of slope

In contrast, RHs showed a moderate correlation between increasing acetate concentration and decreasing burst frequency (*p* = 0.021, *r* =  −0.72, *R*^2^ = 0.52) (Fig. 2A). This relationship did not extend to burst incidence for acetate (*p* = 0.43), propionate (*p* = 0.68) or butyrate (*p* = 0.54), which showed no significant correlations. Increasing acetate concentration was associated with lower resting heart rate in RH (*p* = 0.046, *r* =  −0.54). Sympathetic vascular transduction strongly correlated with acetate concentration in this cohort (*p* = 0.0004, *r* = 0.90) with increasing acetate concentration associated with increasing sympathetic vascular transduction (Fig. 2B).

Despite a relationship between acetate and MSNA burst frequency, systolic blood pressure did not show any notable association with acetate concentrations (*p* = 0.37, *r* =  −022) in patients with resistant hypertension.

## Discussion

These results are the first to demonstrate that increased SCFA concentration in serum, particularly acetate, is associated with lower resting sympathetic drive in patients with resistant hypertension. Moreover, increasing acetate concentration was strongly associated with increasing sympathetic vascular transduction in patients with resistant hypertension, despite chronically elevated adrenergic input to the arterioles. We also report an absence of these findings in healthy controls, indicating a role in sympathetically mediated blood pressure changes in patients with hypertension, which does not appear to be present in the normotensive state.

### Sympathetic modulation via SCFAs: peripheral and central mechanisms

It is well established that sympathetic drive, whether measured by MSNA or noradrenaline spillover, is markedly elevated in patients with resistant hypertension [4, 15, 16]. This represents an important factor in the development and maintenance of the hypertensive state, with sympathetic overdrive not only acting directly to increase total peripheral resistance through arteriolar vasoconstriction [17], but through shifts in blood volume handling and increased cardiac output [18]. As such, direct sympatholysis has become an increasingly common means of reducing blood pressure in this patient cohort, whether pharmacologically [19] or interventionally [20].

However, this work is the first to show a potentially direct modulatory role for SCFA on MSNA. It has been shown previously that much of the effect of SCFA via the gut–brain axis has been via the vagus nerve [21]; however, animal work seems to suggest a direct sympatho-inhibitory role. Mouse work indicates systemic administration of acetate acts to decrease heart rate, and thus mean arterial pressure, which was not blocked by atropine but was inhibited by atenolol [22]. In essence, blocking vagal drive to the heart did not inhibit the response to acetate suggesting that sympathetic withdrawal is the more likely mechanism. Central sympatholytic mechanisms have also been reported. Intracerebroventricular administration of butyrate in spontaneous hypertensive rats results in decreases in mean arterial pressure (MAP) with subsequently diminished activation in the paraventricular nucleus of the hypothalamus—a potential cardioinhibitory control centre [23]. Central mechanisms also extend beyond direct SCFA-receptor interactions within the brain. While certainly autonomic innervation plays a role in this setting, direct SCFA-receptor interactions occur on cardiomyocytes. Direct reductions in acute inotropy is thought to be mediated by *GPCR43* [24], with more chronic changes mediated by SCFA-mediated downregulation of *Egr1*—a cardiovascular regulator gene involved in cardiac hypertrophy [11].

Correlations between elevated acetate concentration and reduced resting heart rate mirror previously reported animal work with acetate supplementation [24]. Likewise, while MSNA is significantly elevated compared with healthy controls as previously reported [17], we saw a correlation between reduced resting sympathetic drive in patients with resistant hypertension and increased serum acetate levels. While changes in heart rate represent a shifting balance of vagal and sympathetic activation at the heart, the vasoconstrictor drive to the skeletal muscle bed is unopposed by parasympathetic innervation.

As such, this reduced MSNA burst frequency with increasing acetate concentration indicates a potential sympatho-inhibitory role of acetate in humans. SCFA target receptors are prominent in sympathetic chain ganglia, particularly *GPR41*, but activation with propionate induces sympathetic activation in vivo and in vitro [25, 26]. GPR43, the primary receptor for acetate, has been localised to the sympathetic chain ganglia in mice [27], with limited functional characterization in terms of sympathetic outflow. As such, whether this receptor–ligand interaction parallels propionate at *GPR4*1 is unknown. Given the conserved homology of these receptors across species, albeit with differences in ligand preference [28], it appears reasonable that this may apply to humans. As such, the results presented here would be most likely indicative of a central sympathoinhibition as the driving force of lower MSNA burst frequency.

Butyrate and propionate are believed to largely signal to the central nervous system (CNS) via *GPR43* within the nodose ganglion of the vagus nerve [29] with subsequent increased vagal afferent firing [30, 31], which may drive the sympatholytic effect. Acetate however is thought to act more directly on the hypothalamus, particularly the arcuate nucleus [32]. Direct projections between the arcuate nucleus and the rostral ventrolateral medulla (RVLM)—the chief sympathetic outflow nucleus within the brainstem—have been indicated by retrograde tracing studies [33]. These neuronal populations however appear to be sparse [34] and as such, an indirect pathway, likely via an axis of the arcuate to paraventricular nucleus to cardiovascular control centres in the medulla appears more likely through gamma-aminobutyric acid (GABA)ergic or neuropeptide Y (NPY) transmission [35]. In effect, this inhibitory signaling via this proposed axis may drive the reduced generation of MSNA bursts via the rostral ventrolateral medulla (RVLM).

### Sympathetic vascular transduction: influence of vasodilatory properties of SCFAs

In our sample, we noted firstly that acetate levels were markedly reduced in patients with resistant hypertension compared to healthy controls. This is unsurprising given previous work indicating patients with essential hypertension had reduced serum acetate levels [14], particularly those with untreated hypertension. The attenuated levels of acetate, but not propionate or butyrate, is consistent in this patient cohort between this work and previous work from our group [14].

Sympathetic vascular transduction is known to be reduced in patients with untreated hypertension [5] and heart failure with reduced ejection fraction [36]. Effectively, this metric represents a reduced capacity to respond to sympathetic stimulus to the vasculature—each MSNA burst is less effective at causing vasoconstriction and a relative rise in diastolic blood pressure as a result. In the setting of hypertension, this is largely thought to be downstream of adrenergic insensitivity [37], which is a product of global sympathetic overdrive, as well as specific vascular remodelling [38]. In essence, the smooth muscle surrounding the arteriole has reached a broadly defined point of maximally generated force and as such, has limited contractile reserve.

It is known that SCFAs may induce vasodilatory effects in an endothelium-dependent manner [39] at SCFA concentrations within the typical physiological range. This has been found to be dependent on GPR41 localised to the vascular endothelium of the resistance vessels. Increased colonic short chain fatty acid production, particularly acetate and butyrate, has been shown to improve vascular function in hypertensive animals [40], which appears to persist in preliminary human trials [13]. With increased vasodilatory stimulus from SCFA signaling, this may offset the vasoconstrictive sympathetic signaling in patients with hypertension. As such, this potentially explains improved sympathetic vascular resistance at higher levels of serum acetate—these patients simply maintain a greater vasoconstrictive reserve to modulate blood pressure. This may extend beyond activation of GPR41 promoting this reduced vascular tone. Acetate has been shown to increase nitric oxide (NO) release, and thus vasodilation, from the endothelium despite elevated levels of angiotensin 2 in rat vascular preparations [41]. The underlying mechanism is potentially independent of the free fatty acid receptors [41].

### Blood pressure does not correlate with acetate concentrations

Within our cohort, serum acetate concentration did not predict automated office blood pressure, with no apparent relationship formed between the two. Given a host of animal work, and burgeoning human trials, supporting the role of SCFA as having antihypertensive effects, this is perhaps surprising. An important consideration in our sample is the role of pharmacotherapy. Previous human work has centred around drug naive patients with largely uncomplicated hypertension [13]. Our patient cohort was multi-drug refractory  and on a significant number of antihypertensive agents with varying degrees of blood pressure control. As such, if this proposed correlation between acetate, or other SCFAs, and blood pressure were direct and observable in this group, it would likely be blunted by the varying degrees of pharmacological interventions present within the sample.

It is worth also considering the multimodal physiology of hypertension in human cohorts, particularly that of patients with resistant hypertension. While we approach a consensus that SCFAs and the microbiome at large are relevant in the maintenance of the hypertensive state, a wide array of other pathophysiological processes underlies the chronic elevation of blood pressure [42]. Fundamentally, decreasing blood pressure via one mechanism tends to lead to blunted antihypertensive responses, hence the growing evidence of the superiority of quadpill formulations of common agents at low doses [43].

### Limitations

Owing to the nature of this study as a cross-sectional analysis, we cannot establish a cause–effect relationship but only draw correlations from our current results. The significant body of evidence from experimental work is in line with our findings and may suggest a causative link.

Clearly, there are significant differences in the age of our cohorts. While hypertension is an age-related condition, and recruitment of age matched controls would prove complex, the age disparity within these samples has the potential to skew the results. While we are not aware of any significant shifts of short-chain fatty acid levels across adulthood, this cannot be ruled out. The microbiome is known to change with advanced ageing [44] and acetate concentration itself is higher in infancy owing to bacterial strains favoured for the role in human milk oligosaccharide digestion [45]. Broadly, however, bacterial composition of the microbiome remains relatively constant through the majority of adulthood [46]. As such, while there are perhaps important caveats to the stability of the microbiome at the extremes of age, for our study, we believe this impact is minimised. We acknowledge however that we may be observing a vascular response with subsequent effects on the sympathetic nervous system as opposed to a sympathetic change with vascular sequalae. It is evident that SCFAs can affect vascular tone and subsequently, this may result in reduced sympathetic stimulus required for the heart to overcome reduced afterload. Regardless, it appears likely that SCFAs have potential cardiometabolic benefit and further research illustrating this mechanism is warranted.

As a result of these differences, we have refrained from direct comparisons of sympathetic vascular transduction between these cohorts given the known age-related changes that occur both in healthy ageing and in the pathophysiology of hypertension.

## Conclusions

This work is the first to compare the sympathetic activity of healthy controls and patients with resistant hypertension alongside short-chain fatty acid levels. We have found that acetate correlates with muscle sympathetic nerve activity as well as sympathetic vascular transduction in patients with resistant hypertension, further supporting the beneficial and cardioprotective role of short-chain fatty acids in cardiovascular disease. Future work should focus on interventions designed to increase the production of SCFAs from the microbiome and how this may directly shift autonomic function in this patient cohort.
